# Elranatamab for Treatment of Multiple Myeloma With Central Nervous Involvement Refractory to Other Interventions

**DOI:** 10.1002/jha2.70203

**Published:** 2026-02-24

**Authors:** Kelley Julian, Briana Peterson, Ghulam Rehman Mohyuddin

**Affiliations:** ^1^ Division of Hematology Huntsman Cancer Institute University of Utah Salt Lake City Utah USA

## Abstract

**Background:**

Plasma cell leukemia and central nervous system involvement are two poor prognostic factors in myeloma, and such patients are often excluded from clinical trials.

**Case:**

A 59‐year‐old patient with primary plasma cell leukemia developed leptomeningeal CNS disease following autologous transplant. Intrathecal chemotherapy and selinexor, pomalidomide, and dexamethasone failed to clear cerebrospinal fluid plasma cells.

**Intervention:**

Elranatamab achieved complete serological and CNS response. The patient recovered from bedbound status to full independence and proceeded to receive CAR‐T therapy. At 8 months post CAR‐T, he remains in sustained MRD negativity.

**Conclusion:**

Sequential bispecific antibody and CAR‐T therapy demonstrates exceptional efficacy in CNS multiple myeloma, offering new hope for this historically terminal condition and supporting the systematic study of novel immunotherapies in high‐risk myeloma populations.

**Trial Registration**: The authors have confirmed clinical trial registration is not needed for this submission.

1

Historically, patients with multiple myeloma involving the central nervous system (CNS) have not benefited from recent therapeutic advances [1]. The prognosis for such patients has remained dire, and many currently approved agents do not have CNS penetration [2]. Furthermore, CNS involvement is often an exclusion criterion for clinical trials, limiting treatment options and complicating extrapolation of clinical trial results to this patient population [3]. The newest immunotherapies in myeloma—chimeric antigen receptor therapy (CAR‐T) and bispecific therapy have also excluded patients with CNS disease from pivotal trials [3]. Similarly, those with plasma cell leukemia, another population in need for novel agents, are often excluded from these trials [3].

As a result, efficacy of these novel agents in patients with CNS disease remains unknown. A case series of 10 multiple myeloma patients with CNS disease receiving BCMA‐directed CAR‐T demonstrated a modest progression free survival of 6.3 months, and an overall survival of 13.3 months [4]. Another series of nine patients treated with bispecifics for CNS disease (eight of which received talquetamab, one of which received teclistamab) showed that six patients had responses in their CNS, but there was limited follow‐up to ascertain durability [5].

We highlight here an exceptional case report in which both CAR‐T and bispecific therapy were used for an enduring, ongoing response in a patient with CNS disease and primary plasma cell leukemia that had proven refractory to other interventions.

This is a retrospective chart review of a single patient who provided written consent for the review of his medical records and publication of these results. Claude was used to visually enhance the initial draft of Figure 1, with all text provided by the authors.

**FIGURE 1:**
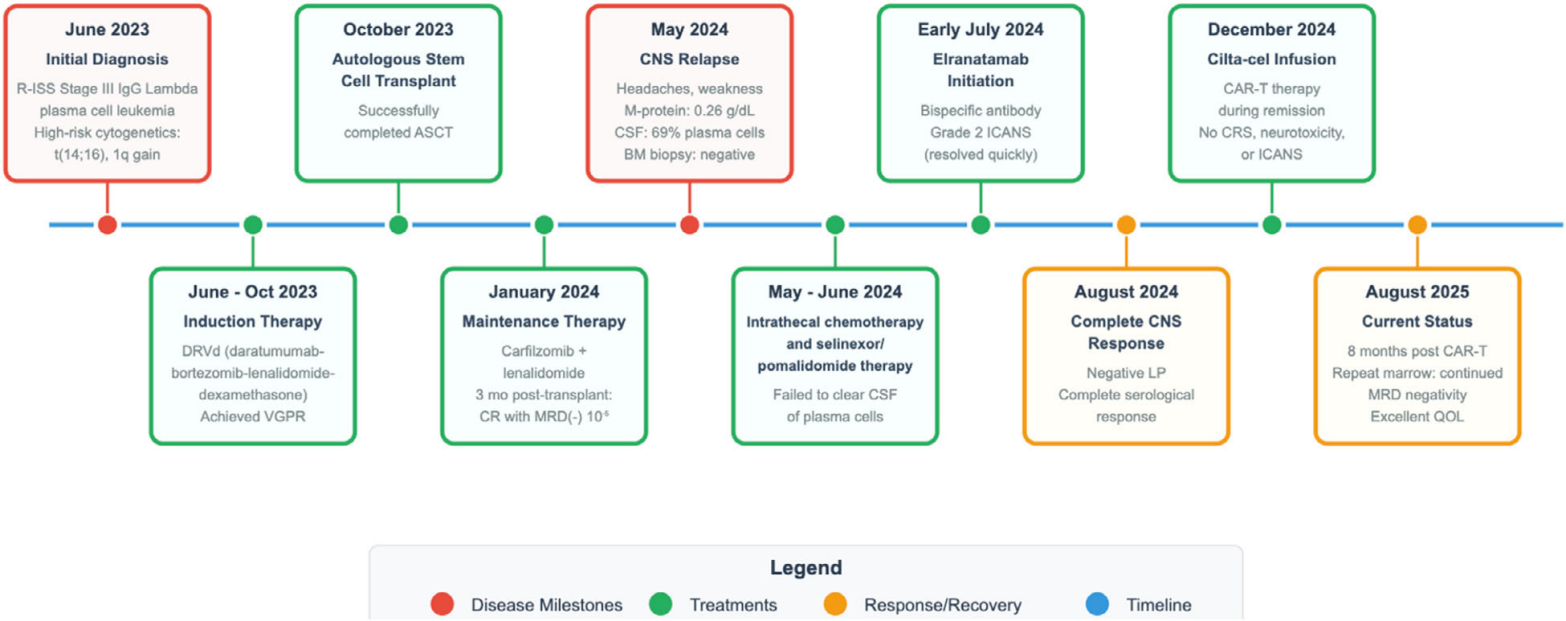
Timeline depicting the patients disease course.

A 59‐year‐old patient was diagnosed with R‐ISS Stage III IgG primary plasma cell leukemia in June 2023 after being evaluated for months of back pain. Cytogenetics confirmed high risk disease with translocation (14;16) and 1q gain. Patient received daratumumab‐bortezomib‐lenalidomide‐dexamethasone (DRVd) as induction therapy, achieving a very good partial response, and then in October 2023 received autologous stem cell transplant. At 3 months post‐transplant, a bone marrow biopsy showed no evidence of disease on an in‐house flow cytometry panel (10^−5^) and serum markers indicated a minimal residual disease (MRD) negative complete response. He was then placed on maintenance with carfilzomib and lenalidomide in January 2024 and remained on this until May 2024, at which point he developed headaches and progressive upper and lower extremity weakness. His monoclonal protein was now detectable at 0.26 g/dL. Leptomeningeal CNS disease was confirmed with brain and spinal MRI and lumbar puncture, which showed 69% plasma cells in the cerebrospinal fluid (CSF) in May 2024. His repeat bone marrow biopsy at that time remained negative for clonal plasma cells on flow cytometry. Over the course of the next month, the patient received five intrathecal chemotherapy administrations with methotrexate, cytarabine, and hydrocortisone, all of which failed to clear his CSF of plasma cells. The patient was collected for cilta‐cel at the end of June, and subsequently selinexor‐pomalidomide‐dexamethasone was briefly started as bridging while awaiting CAR‐T manufacturing and infusion. His condition rapidly declined during this period, his weakness profoundly worsened with an eventual ECOG of four, until the point where the patient became completely bedbound, experienced intense nausea, and had > 40 pounds of weight loss. After less than a month of this bridging therapy with concurrent intrathecal chemotherapy, the decision was made to abort this regimen and start elranatamab for his CNS disease, which he began this in early July 2024.

The patient underwent ramp‐up while admitted and experienced Grade 2 Immune Effector Cell‐Associated Neurotoxicity Syndrome (ICANS) after the first dose, characterized initially as Grade 1 with worsening to Grade 2 given an ICE score of 6 with worsening somnolence, which promptly resolved with dexamethasone. The remainder of his ramp‐up was uneventful and with extensive rehabilitation, this patient's performance status slowly and steadily improved until he regained full physical independence, approximately 3 months post discharge with an ECOG of one. A complete serological and CNS response was achieved within the first two cycles of therapy with a repeat negative lumbar puncture in August 2024. In December 2024 to utilize previously collected cells before the manufacturer expiration, the patient opted to receive cilta‐cel. His course remained uncomplicated, with no events of CRS, neurotoxicity or ICANS to date. The bone marrow biopsy 3 months out showed MRD negativity both on flow and adaptive (10^−6^) testing, as well as a negative PET/CT. As of December 2025, the patient is 12 months beyond CAR‐T infusion and has dealt with intermittent mild cytopenias that are currently stable and recovered, but otherwise myeloma markers remain negative, and he has returned to a fully functional status and excellent quality of life. A repeat bone marrow biopsy 8 months post CAR‐T infusion continues to show MRD negativity. The patient's disease course is summarized in Figure 1.

This case report highlights how the use of novel immunotherapies produced an ongoing exceptional response in multiple myeloma that was otherwise refractory to therapies for CNS disease and historically has been associated with a terminal prognosis [2]. Four key educational lessons can be learned.

First, although leukapheresis had been performed and cilta‐cel had been collected, a dramatic worsening in clinical status occurred during the period while delivery was awaited. If access to bispecific therapy had not been available, it is likely that the patient would not have survived the bridging period. This exemplifies how the logistical delays in accessing CAR‐T can prevent patients with rapidly progressing disease from receiving it when they need it most [6, 7]. Use of off‐the‐shelf allogeneic CAR‐T products may help alleviate this in the future [8].

Second, bispecifics can immediately be deployed—even in patients with CNS disease and rapidly worsening clinical status—underscoring their clinical utility, ease of access, and delivery in otherwise extreme situations. A caveat is that this patient was treated at an academic center with access to multidisciplinary advanced care, but as community hospitals gain further familiarity with bispecific therapy and protocols for outpatient ramp‐up come into effect, it will be increasingly likely that bispecifics can be deployed urgently to patients who need them.

Third, a “one size fits all” sequencing approach does not work for patients with myeloma. Although current guidelines recommend utilizing CAR‐T before bispecifics [9], these guidelines are not based on randomized data of various sequencing approaches, but rather on retrospective data. Patients who get CAR‐T first (as opposed to bispecifics) are different than those whose disease was not stable enough to receive CAR‐T first. Although, there is a rationale pertaining to T‐cell fitness that makes a case for reserving bispecifics after CAR‐T [10], there are situations where bispecifics have to be given first. Furthermore, an arbitrary 9‐month expiration policy from the time of collection to when cilta‐cel has to be administered, currently exists from the manufacturer of cilta‐cel. The impending expiration influenced our decision to administer cilta‐cel. Further work is needed to determine whether the current expiration policy reflects is influenced by commercial concerns or by a scientific rationale.

Fourth, CAR‐T during remission carries nuanced risk‐benefit decisions. Cilta‐cel was given to avoid expiration of collected cells and to consolidate remission. However, in the setting of deep response to bispecific therapy, the added benefit of CAR‐T remains uncertain. The administration of cilta‐cel is not without risk, and such decisions have a risk‐benefit profile that is hard to fully ascertain, given late onset neurological toxicities and secondary cancers seen with cilta‐cel.

Other lessons that can be learnt from this case include that the acquisition of MRD negativity after initial induction therapy, as was seen in our case can be short‐lived, and decisions regarding de‐escalation of therapy based on early MRD achievement, in such a high‐risk patient population, should be made with caution. This patient population should be prioritized for evaluation of novel maintenance approaches. It is also unclear how BCMA bispecifics as bridging compare to the use of non‐BCMA bispecifics such as talquetamab. Whereas there is currently great interest in talquetamab as bridging [11], it is unclear if a brief duration of bridging therapy with BCMA bispecifics would lead to an antigen loss, and whether BCMA bispecifics could be used instead of talquetamab in this situation. The decision to pursue elranatamab as opposed to a GPRC5D bispecific such as talqeutamab was based on tolerability concerns, and a desire to avoid the oral toxicity and weight loss in this patient who was in a nearly terminal condition. It is unknown how the outcomes would have been if talquetamab was given instead, but we speculate it could have worked as well. Given the limitations of this single patient review, it is unclear how this doublet BCMA strategy minimizes antigen loss/modification compared to a sequential strategy of targeting different receptors. This should be the subject of future prospective research.

In summary, the sequential use of elranatamab and cilta‐cel in this case led to an exceptionally deep and enduring remission, highlighting the potential of these agents to treat two historically difficult‐to‐treat subsets of myeloma: plasma cell leukemia and those with CNS disease. We continue to advocate for systematic studies of bispecific antibodies in high‐risk myeloma populations, such as the patient we have presented here, to establish more concrete and evidence‐based treatment protocols in this aggressive disease.

## Author Contributions

K.L.J., B.R, and G. R.M. conceptualized the work and prepared the draft. All authors were involved in the care of the patient.

## Funding

Ghulam Rehman Mohyuddin reports honoraria from Medscape and MashupMD for writing. His institution has received research funding from Janssen. Kelley Julian reports advisory/consultancy for Pfizer, Janssen, and Sanofi.

## Ethics Statement

The authors have nothing to report.

## Conflicts of Interest

The authors declare no conflicts of interest.

## Data Availability

The authors have nothing to report.
